# 514. The Impact of Xeruborbactam on *in vitro* Activity of Cefiderocol against a Panel of *Acinetobacter baumannii* Enriched in Isolates with Reduced Cefiderocol Susceptibility

**DOI:** 10.1093/ofid/ofae631.166

**Published:** 2025-01-29

**Authors:** Takafumi Hara, Naoki Ishibashi, Dai Miyagawa, Motoyasu Onishi, Olga Lomovskaya, Yoshinori Yamano

**Affiliations:** Shionogi & Co., Ltd., Toyonaka, Osaka, Japan; Pharmaceutical Research Division, Osaka, Osaka, Japan; Shionogi & Co., Ltd., Toyonaka, Osaka, Japan; Shionogi & Co., Ltd., Toyonaka, Osaka, Japan; Qpex Biopharma Inc., San Diego, California; Shionogi & Co., Ltd., Toyonaka, Osaka, Japan

## Abstract

**Background:**

Cefiderocol (CFDC) is a siderophore cephalosporin with potent activity against resistant gram-negative bacteria. Recent SIDERO-WT surveillance studies demonstrated potent activity of CFDC against 5,225 isolates of *Acinetobacter baumannii* complex (ACB) (MIC_50_/_90_=0.12/1 µg/mL; 96% susceptible using CLSI breakpoint) including 2,810 carbapenem-resistant isolates (MIC_50_/_90_=0.25/2 µg/mL; 94% susceptible). CFDC MIC values were higher (MIC_50_/_90_=4/8 µg/mL) against metallo-β-lactamase (MBL) producers (N=25 including 21 NDM producers; 0.48% of total ACB) and isolates that produced PER or VEB β-lactamase (N=103; 1.98% of total ACB; MIC_50_/_90_=128/256 µg/mL). Xeruborbactam (XER) is a novel beta-lactamase inhibitor that inhibits multiple serine-type β-lactamases (SBLs) and MBLs. In this study, we evaluated the combination of CFDC and XER against a challenge panel of ACB enriched with CFDC-resistant isolates.

MIC50/90 of cefiderocol (CFDC) alone and in the presence of xeruborbactam (XER) against Acinetobacter baumannii

**Figure:**
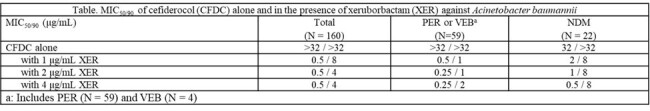

**Methods:**

A challenge panel of 160 ACB isolates that included 59 PER- or VEB-producing and 22 NDM-producing isolates was used for this study. 144 (90%) and 136 (85%) isolates had CFDC MIC > 1 µg/mL (FDA non-susceptible breakpoint) and > 2 µg/mL (EUCAST non-susceptible PK/PD breakpoint), respectively. β-lactamases content was determined by PCR or whole genome sequencing. MICs were determined for CFDC alone and in combination with 1, 2 and 4 μg/mL of XER according to CLSI guidelines for CFDC using iron-depleted CAMHB.

**Results:**

MIC_50_/MIC_90_ of CDFC alone and XER alone against 160 ACB were > 32/ > 32 and 16/ > 16 μg/mL, respectively. The addition of xeruborbactam at all concentrations resulted in a reduction of CFDC MICs. For the subset of 59 highly CFDC-resistant PER or VEB producers, XER at ≥ 2 µg/mL reduced CFDC MIC_50_/_90_ from > 32/ > 32 to 0.25/1 µg/mL, which is similar to MIC_50_/_90_ values observed for CFDC alone for all ACB from recent surveillance studies. XER at 4 μg/mL reduced MIC_50_/_90_ of NDM producers from 32/ > 32 μg/mL to 0.5/8 μg/mL.

**Conclusion:**

Addition of XER significantly improved the activity of CFDC against isolates of ACB with elevated CFDC MICs.

**Disclosures:**

**Takafumi Hara, MSc**, SHIONOGI & CO., LTD.: Employee **Naoki Ishibashi, MD**, SHIONOGI & CO., LTD.: Employee **Dai Miyagawa, n/a**, SHIONOGI & CO., LTD.: Employee **Motoyasu Onishi, PhD**, Shionogi & Co., Ltd.: Employee **Olga Lomovskaya, PhD**, Qpex Biopharma Inc.: Board Member|SHIONOGI & CO., LTD.: Employee **Yoshinori Yamano, PhD**, Shionogi & Co., Ltd.: Employee

